# The Predictive Value of Salt Sensitivity and Osmotic Fragility in the Development of Cerebral Small Vessel Disease

**DOI:** 10.3390/ijms21062036

**Published:** 2020-03-16

**Authors:** Larisa A. Dobrynina, Alla A. Shabalina, Kamila V. Shamtieva, Elena V. Gnedovskaya, Alexander B. Berdalin, Marina V. Krotenkova

**Affiliations:** 1 Research Center of Neurology, 80 Volokolamskoe shosse, 125367 Moscow, Russia; ashabalina@yandex.ru (A.A.S.); kamila.shamt@gmail.com (K.V.S.); gnedovskaya@mail.ru (E.V.G.); krotenkova_mrt@mail.ru (M.V.K.); 2 Federal State Budgetary Institution “Federal Center for Cerebrovascular Pathology and Stroke”, 1, stroenie 10, Ostrovityanova, 117342 Moscow, Russia; alex_berdalin@mail.ru

**Keywords:** cerebral small vessel disease, glycocalyx, Na^+^/K^+^-ATPase, osmotic fragility, salt sensitivity, white matter hyperintensities

## Abstract

Increased salt intake in food probably affects the progression of cerebral small vessel disease (CSVD), which justifies the study of disturbances in sodium homeostasis associated with the development of CSVD. We aimed to clarify the role of salt sensitivity and osmotic fragility in the development of CSVD. Erythrocyte salt sensitivity was measured using the modified salt blood test, and osmotic fragility was measured using the classic osmotic fragility test in 73 patients with CSVD (48 women; 60.1 ± 6.5 years) and 19 healthy volunteers (14 women; 56.9 ± 6.4 years). Salt sensitivity and osmotic fragility exhibited a predictive value in relation to CSVD. These parameters were associated with an increase in white matter hyperintensities (*p* = 0.019 and 0.004, respectively). Their simultaneous use increased their predictive ability for CSVD (*p* < 0.000001; AUC (95% CI), 0.824 (0.724–0.923)). The possibility of predicting CSVD using erythrocyte salt sensitivity and osmotic fragility indicates the value of the individual glycocalyx buffer capacity in relation to sodium and the activity of sodium channels in the development of CSVD. Increased salt sensitivity and osmotic fragility seem to be risk factors for CSVD.

## 1. Introduction

Cerebral small vessel disease (CSVD), which is associated with age and vascular risk factors, is the main cause of vascular cognitive impairment and mixed variants of Alzheimer’s disease and is a significant cause of stroke, disability and mortality [1]. Arterial hypertension (AH) is the main risk factor for age-related CSVD [1,2,3]. However, in a large proportion of cases, there is no direct causal relationship between AH and CSVD, which indicates that other conditions, independent or comorbid with AH, are involved in the development of the disease. One of them may be the disturbance in sodium homeostasis, mainly considered as a phenomenon of salt sensitivity. The latter implies an increase in blood pressure with excessive salt loading and a decrease in blood pressure at low salt diet in hypertensive and normotensive patients [4]. This assumption stems from the association between increased salt intake and hypertension-independent cardiovascular risk [5], the accelerated development of CSVD in stroke-prone spontaneously hypertensive rats [6] and increased areas of white matter hyperintensities (WMHs) [7,8] and other MRI signs of CSVD, such as lacunes and microbleeds, after adjustment for age and AH [8].

One of the ways that disturbances in sodium homeostasis may have a direct effect on the development of CSVD is the increase in blood–brain barrier (BBB) permeability. This is indirectly supported by the established link between hypernatremia and increased brain volume, which was accompanied by increased contrast transit time in unchanged white matter on MRI T1-weighted images [7]. It is likely that the buffer capacity of endothelial and red blood cell glycocalyces relative to sodium is the determining factor in the relationship of dietary sodium with plasma sodium and BBB permeability. Salt consumption that exceeds the ability of the negatively charged glycosaminoglycans of the glycocalyx to retain sodium is associated with damage to this network and the release of Na^+^ through endothelial sodium channels, beyond the vascular bed [9,10]. Several studies have shown that increased BBB permeability is associated with endothelial glycocalyx damage and disruption of sodium transporters, mainly Na^+^/K^+^-ATPase [11,12,13,14].

Furthermore, Na^+^/K^+^-ATPase expression in the vascular endothelium is an important regulator of endothelial stiffness under an excessive salt load [15].

Experimental and clinical data suggest that CSVD development in individuals with increased salt intake may depend on the individual characteristics of the glycocalyx and the functioning of sodium transporters at the cell membrane. We used the modified salt blood test proposed by H. Oberleithner et al. [16,17] to determine individual salt sensitivity by measuring the buffer capacity of a patient’s red blood cell glycocalyx. This method is based on the functional unity of endothelial and red blood cell glycocalyces and involves measuring the buffer capacity of the preshielded red blood cell glycocalyx in a plasma-free medium, based on their sedimentation rate in solutions with different sodium chloride molarities [16,17].

To describe the functional properties of sodium transporters at the cell membrane, we chose erythrocyte osmotic fragility, which corresponds to erythrocyte ability to withstand hemolysis in hypotonic solutions [18]. The osmotic fragility test was carried out by assessing the resistance of red blood cells to lysis in hypotonic solutions with decreasing concentrations, followed by the sequential measurement of the degree of hemolysis using absorption spectrometry [18]. The link between osmotic fragility and sodium transporter function, in particular, Na^+^/K^+^-ATPase, was first established in sickle cell anaemia [19,20]. The osmotic fragility test is still used for the diagnosis of hereditary disorders of the red blood cell membrane [21,22]. The osmotic fragility test was also chosen because of data obtained from young spontaneously hypertensive rats—increased red blood cell permeability in relation to Na^+^ and K^+^ [23] and the accumulation of red blood cells in capillaries and arterioles [24] long before the occurrence of changes in the brain and blood vessels.

### Aim

To clarify the value of salt sensitivity and osmotic fragility in the development of CSVD.

## 2. Results

The main demographic data and risk factors of the study group with CSVD and the control group are presented in Table 1. Both groups had a female predominance. There was a significant difference between the patients with CSVD and the subjects in the control group regarding the presence and severity of AH and diabetes mellitus (DM) type 2.

The main clinical symptoms and MRI signs of CSVD are presented in Table 2.

**Figure 1:**
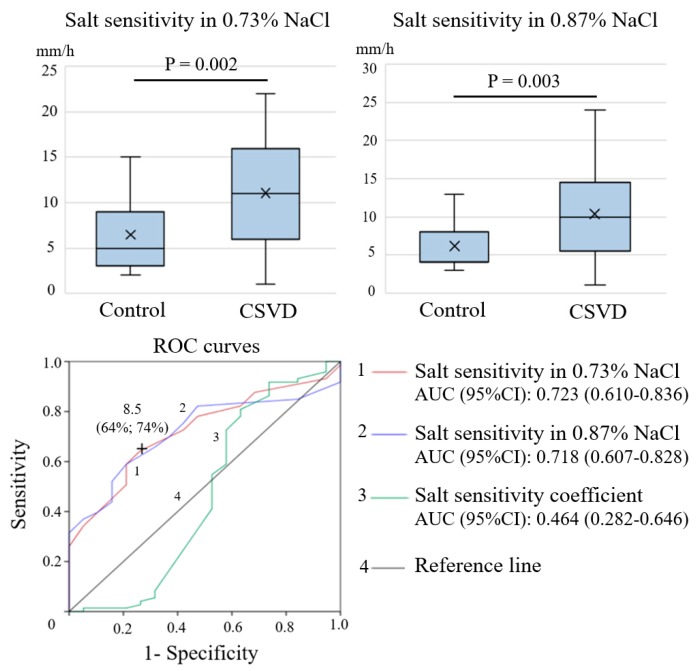
Comparison of the salt sensitivity parameters between patients with CSVD and the control group. Receiver operating characteristic (ROC) curves for salt sensitivity tests relative to CSVD development are shown.

Patients with CSVD exhibited a significantly higher salt sensitivity in 0.73% and 0.87% sodium chloride solutions (*p* = 0.002 and *p* = 0.003, respectively) compared with the control group (Figure 1). The salt sensitivity coefficient did not differ significantly between the groups (*p* = 0.067). Laboratory tests of salt sensitivity were used to determine the most significant predictor of the development of CSVD. According to the results of the receiver operating characteristic (ROC) analysis, the largest area under the curve (AUC) was the salt sensitivity in 0.73% sodium chloride solution (AUC (95% CI), 0.723 (0.610–0.836)) (Figure 1), and its threshold value of 8.5 mm/h yielded the best characteristics with a sensitivity of 64% and a specificity of 74% (Figure 1).

**Figure 2:**
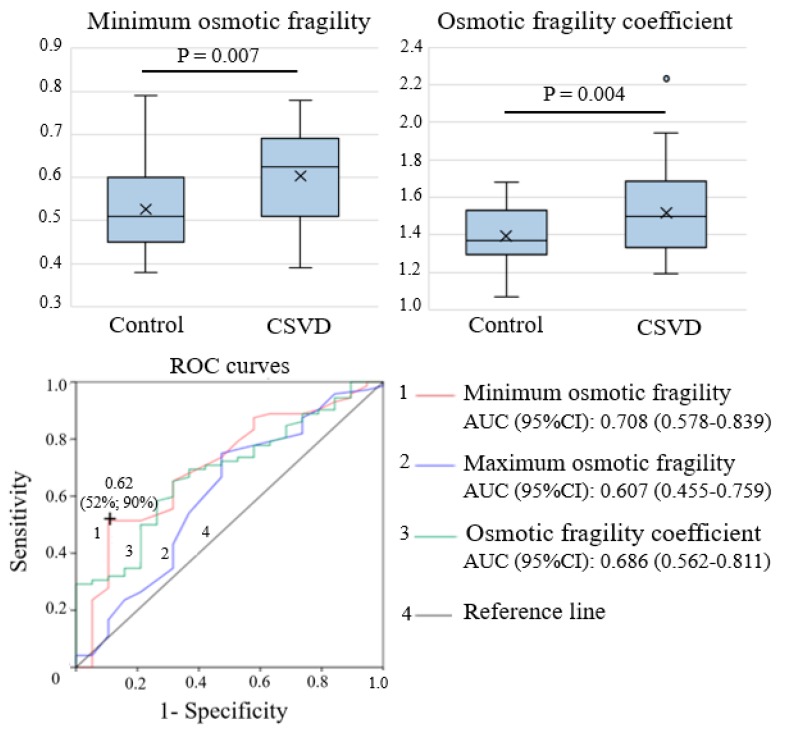
Comparison of the osmotic fragility parameters between patients with CSVD and the control group. ROC curves of osmotic fragility parameters in relation to CSVD development are shown.

Compared with the control group, patients with CSVD had a significantly higher minimum osmotic fragility and coefficient of osmotic fragility (*p* = 0.007 and *p* = 0.004, respectively); however, no significant differences were observed for maximum osmotic fragility (*p* = 0.169). The ROC analysis showed that, among the laboratory tests used for the determination of osmotic fragility, the minimum osmotic fragility had the highest predictive ability for the development of CSVD (AUC (95% CI), 0.708 (0.578–0.839)), and its threshold value of 0.62 yielded the best characteristics with a sensitivity of 52% and a specificity of 90% (Figure 2).

The salt sensitivity and osmotic fragility parameters were not interlinked.

**Figure 3:**
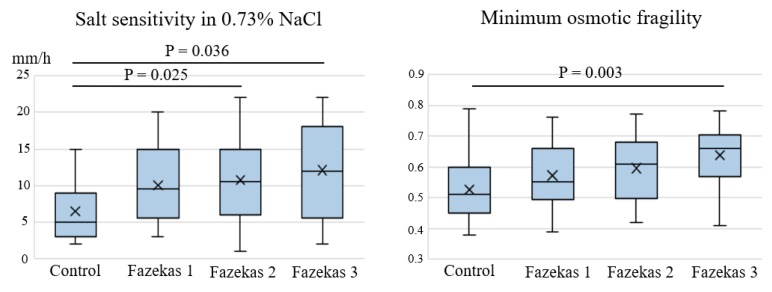
Association between salt sensitivity, osmotic fragility and the severity of WMHs depending on the Fazekas grade.

The tests with the best predictive ability for CSVD—salt sensitivity in 0.73% sodium chloride solution and minimum osmotic fragility—were compared with MRI signs of CSVD. Salt sensitivity in 0.73% sodium chloride solution and minimum osmotic fragility exhibited significant differences depending on the severity of WMHs according to the Fazekas grades (*p*, ANOVA = 0.019 and 0.004, respectively). A subsequent intergroup comparison revealed a significant increase in salt sensitivity in patients with Fazekas grades 2 and 3 compared with the controls and in osmotic fragility in patients with Fazekas grade 3 compared with the controls (Figure 3).

There were no significant associations between salt sensitivity/osmotic fragility and the severity of other MRI signs of CSVD, such as lacunes, microbleeds and enlarged perivascular spaces.

Data on salt sensitivity in 0.73% sodium chloride solution and minimum osmotic fragility were used to construct a predictive model of CSVD development using binary logistic regression. Its characteristics are shown in Table 3.

The resulting logistic regression model is described in the second step of the step-by-step algorithm for including predictors. The combined tests for the model coefficients show its high statistical significance *p* < 0.000001 (test statistics = 23.649 – χ^2^ test). The –2log likelihood value was 70,059 in the second step. The R2 coefficient of determination is 35.1% of the variance of the dependent variable, explained by factors included in the model.

According to the Hosmer–Lemeshow goodness-of-fit test, the predictive model based on these two predictors makes it possible to successfully classify cases as part of dividing the sample into 10 levels (*p* = 0.544). 

In accordance with the model of binary logistic regression, the predicted probability of CSVD development (*P_CSVD_*) is calculated using the equation: *P_CSVD_ = 1* – $\frac{1}{1+e^{-z}}$, where *e* is the base of the natural logarithm, *z* is a linear function and equal *constant + B_1_ × χ _1_ + B_2_ × χ_2_*_,_ where *χ*_1_ is salt sensitivity in 0.73% sodium chloride solution and *χ*_2_ is minimum osmotic fragility, while *B_1_* and *B_2_* are coefficients of salt sensitivity in 0.73% sodium chloride solution and minimum osmotic fragility, respectively.

**Figure 4:**
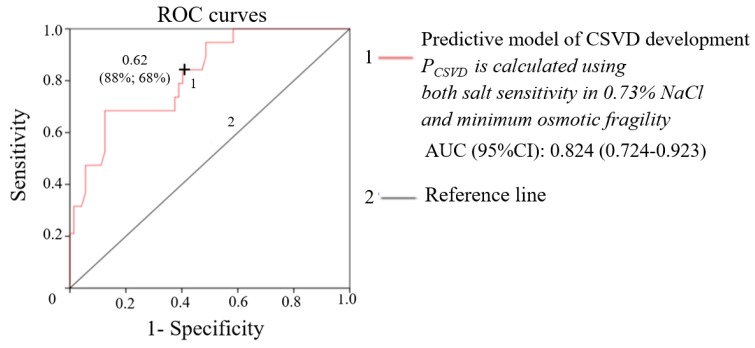
ROC curve of the predictive model of CSVD development.

The predictive model of CSVD exhibited better characteristics when the two tests were used together in the equation, rather than separately (AUC (95% CI): 0.824 (0.724–0.923)). According to the ROC analysis, the threshold value of *P_CSVD_* was 0.62, the sensitivity was 88% and the specificity was 68% (Figure 4).

## 3. Discussion

To clarify the potential role of disturbances in sodium homeostasis in the development of CSVD, the modified salt blood test [16,17] and the osmotic fragility test [18] were used to measure the parameters of salt sensitivity and osmotic fragility in the erythrocytes of patients with CSVD. Their sensitivity and specificity in predicting CSVD and their correlation with MRI signs were also assessed.

The tests used in this study reflect the events that occur when dietary salt is consumed: the filling of the glycocalyx with sodium according to its buffer capacity and the movement of sodium mediated by endothelial sodium channels into the intercellular space, via the activity of sodium transporters, mainly Na^+^/K^+^-ATPase [9,10]. These tests were chosen based on the measurable parameters of BBB permeability, which is the leading mechanism of CSVD development [25,26]. It was found that damage to the endothelial glycocalyx and disruption of the sodium transporters at the cell membrane were associated with increased BBB permeability [11,12,13,14]. The consumption of salt amounts that exceed the buffer capacity of the glycocalyx is a factor that contributes to its destruction and to the movement of sodium from the vascular bed into the intercellular space [9,10]. It was shown that the expression of Na^+^/K^+^-ATPase and the related endothelial stiffness increase under a high salt load [15]. The use of erythrocyte models to estimate individual salt sensitivity based on the sodium buffer capacity of the glycocalyx and of osmotic fragility based on the activity of sodium transporters is justified by the similarity between the endothelium and erythrocytes in relation to sodium transporters [27] and by the functional similarity of their glycocalyces [16,17]. Previously, the salt blood test was used only in healthy volunteers and was recommended as an in vitro test to evaluate salt sensitivity in the prevention and treatment of vascular dysfunction [16,17]. To determine the functional properties of cell-membrane sodium transporters, an osmotic fragility test was used, which, as mentioned previously, is a classic test in the diagnosis of several hereditary diseases of red blood cell membranes [21,22]. This test has not previously been used in patients with CSVD. It should be noted that sickle cell anaemia is currently associated with CSVD development, as well as endothelial dysfunction and the formation of WMHs [28,29].

The tests used in this study, which reflect different pathogenetic mechanisms, showed unidirectional results—the possibility of predicting CSVD based on a diagnosis using MRI signs and clinical manifestations. ROC analysis was used to identify the parameters of the two tests that were most closely related to CSVD development—salt sensitivity in 0.73% sodium chloride solution and minimum osmotic fragility. The salt sensitivity in 0.73% sodium chloride solution and minimum osmotic fragility values were not intercorrelated, which indicates the independence of these parameters and the different functional capabilities of the glycocalyx and sodium transporters during increased salt consumption. This is consistent with the equivalence between salt sensitivity and osmotic fragility in predicting CSVD when their threshold values are exceeded, which was established in this study. Concomitantly, the use of the two tests in the predictive model of CSVD increased the likelihood of diagnosis. This indicates the pathological synergistic contribution of increased salt sensitivity and osmotic fragility to the development of CSVD.

The predictive ability established here was verified by clarifying the relationship between salt sensitivity in 0.73% sodium chloride solution or minimum osmotic fragility with the MRI signs of CSVD. Significant differences in increased salt sensitivity and osmotic fragility were detected only relative to the severity of WMHs, according to the Fazekas grades. WMHs are a pathogenetically heterogeneous sign of CSVD [30]. Until recently, they were considered to be exclusively post-ischaemic changes caused by atherosclerosis [31]. Multimodal studies have determined the importance of increased BBB permeability in the formation of WMHs [25,26]. In a recent study, Heye et al. (2016) found that hypernatremia was involved in this mechanism in the form of a sodium-dependent increase in brain volume and an increased contrast transit time in unchanged white matter as measured using MRI T1-dynamic contrast. The results of our study, which found an association between increased salt sensitivity and osmotic fragility and WMH but not lacunes of ischaemic origin, indicate the importance of the assessed parameters in BBB permeability. This is consistent with previously established data on the role of endothelial glycocalyx damage and disruption of sodium transporters, mainly Na^+^/K^+^-ATPase, in the increase in BBB permeability [11,12,13,14]. Our data differ from the results of the study of Makin et al. (2017), who reported an association between hypernatremia and both WMHs and lacunes. This may be explained by the significant effect of hypernatremia on the development of atherosclerosis or differences in disease severity and duration. It should be noted that our evidence-based discussion of the role of the studied parameters in BBB permeability is supported by information regarding the location of Na^+^/K^+^-ATPase on the endothelial cell membrane facing the brain surface, which ensures the passage of three sodium ions into the cerebrospinal fluid in exchange for the passage of two potassium ions into the cell [11,12].

In conclusion, this study used tests for determining salt sensitivity and osmotic fragility in the red blood cells of patients to establish the possibility of predicting CSVD using individual threshold values of salt sensitivity in 0.73% sodium chloride solution and minimum osmotic fragility, with achievement of a greater diagnostic accuracy when the two parameters were measured at the same time. This indicates the importance of the functional capacity of the glycocalyx relative to sodium and the activity of membrane sodium transporters in the development of CSVD in individuals with increased salt intake. Moreover, the present findings allow us to view salt sensitivity and osmotic fragility as risk factors for CSVD. Further research is needed to clarify the ideal conditions for the application of these data.

## 4. Materials and Methods

This study included patients aged 46–70 years who presented with cognitive complaints at the Research Centre of Neurology (Moscow, Russia) between January 2016 and December 2017 and whose brain changes on MRI corresponded to CSVD (lacunes, WMHs, enlarged perivascular spaces, microbleeds and cerebral atrophy) [32]. Patients with Fazekas grade 1 WMHs were included in the study if they had AH stage 2 or 3 and/or ≥1 lacune.

### Exclusion Criteria

The exclusion criteria were as follows: (1) severe dementia; (2) cognitive impairments caused by probable Alzheimer’s disease according to the National Institute on Aging (United States) criteria [33,34]; (3) patients with small subcortical infarcts/lacunes <3 months after an acute cerebrovascular event; (4) CSVD caused by other independent causes (genetic, inflammatory, thrombophilic, systemic or toxic causes or a history of severe migraines); (5) a different cause of stroke and a concomitant brain pathology other than CSVD; (6) >50% atherosclerotic stenosis of the extra- or intracranial arteries; (7) a serious medical condition—e.g., cardiac disorder (ejection fraction <50%), endocrine condition (DM type 1 or 2 with severe vascular complications), uncompensated thyroid disorder or renal condition (chronic kidney disease with glomerular filtration rate <30 mL/min) and (8) contraindications to MRI studies.

The control group consisted of volunteers with no clinical or MRI evidence of vascular or degenerative brain pathology who were matched for age and gender. In accordance with the above criteria, the study included 73 patients (48 women; average age 60.1 ± 6.5 years) and 19 healthy volunteers (14 women; average age 56.9 ± 6.4 years). The study was approved by the Local Ethics Committee of Research Centre of Neurology (Moscow, Russia). The ethics statement number is 2–4/16 dated 17 February 2016. All subjects signed an informed consent form for participation in the study and for processing of their personal data.

The presence of classic vascular risk factors (AH [35], DM type 2, hypercholesterolemia, obesity and smoking) was assessed in the patients and in the controls.

MRI data were acquired using a Siemens MAGNETOM Verio 3T scanner (Siemens Medical Systems, Erlangen, Germany) with a standard 12-channel matrix head coil. To evaluate STRIVE criteria [32], the patients and control individuals underwent axial spin-echo T2-weighted imaging (TR, 4000 ms; TE, 118 ms; slice thickness, 5.0 mm; in-plane resolution, 1.5 mm^2^; duration, 2 min 02 s); sagittal 3D T2 FLAIR (TR, 6000 ms; TE, 395 ms; isotropic voxel, 1 × 1 × 1 mm^3^; duration, 7 min 12 s); sagittal 3D Т1-mpr (TR, 1900 ms; TE, 2.5 ms; isotropic voxel, 1 × 1 × 1 mm^3^; duration, 4 min 16 s); diffusion-weighted imaging (DWI) using an axial spin-echo echo-planar imaging sequence with two b-values (0, 1000 s/mm^2^) (TR, 4000 ms; TE, 100 ms; slice thickness, 4 mm; duration, 1 min 20 s) and axial-susceptibility-weighted imaging (SWI) sequence with magnitude and phase image reconstruction (TR, 28 ms; TE, 20 ms; slice thickness, 1.2 mm; FOV, 179 × 230 mm^2^; duration, 8 min 12 s).

Two neuroradiologists (E.K. and B.A.) who were blinded to the clinical information evaluated the brain MRI studies in a standardized manner. No STRIVE criteria were found in the control group. There were no acute or recent small lacunar infarcts based on DWI analysis in the patient group. The Fazekas scale was used to quantify T2 FLAIR WMHs (grades 0–3) [36]. White matter and basal ganglia lacunes were graded on T2 FLAIR images depending on their number (<5, 5–10 and >10 lacunes). Microbleeds were rated on SWI images depending on their number (<5, 5–10 and >10) in the basal ganglia and frontal, parietal, occipital and temporal lobes separately. The perivascular spaces were graded based on their size (1–4 mm) in the centrum semiovale and basal ganglia.

Salt sensitivity was evaluated using the modified salt blood test [16,17]. The modification consisted of replacing specialized buffers such as HEPES and albumin with 3% dextran, which has similar functional properties. Blood was obtained from the cubital vein in the morning and on an empty stomach, placed in Vacutainer blood collection tubes containing K3 EDTA and centrifuged for 5 min at 1800× *g*. The supernatant was removed and 80 µL of the residual liquid (containing the erythrocyte mass) was pipetted into two test tubes, and 3% dextran was added to each tube. A solution of 0.73% sodium chloride (0.73% NaCl, 120 µL) was added to the first test tube, and a solution of 0.87% sodium chloride (0.87% NaCl, 120 µL) was added to the second tube. The resulting suspensions were incubated for 60 min at room temperature in capillary tubes, after which the erythrocyte sedimentation rate (in mm/h), the respective salt sensitivity in 0.73% NaCl and 0.87% NaCl solutions, and the ratio of these two parameters (which was used as the salt sensitivity coefficient) were measured.

The function of cell-membrane sodium transporters was evaluated using the osmotic fragility test [18]. Blood was obtained by cubital venepuncture in the morning and on an empty stomach and placed in Vacutainer blood collection tubes containing K3 EDTA. Subsequently, 20 µL of whole blood was pipetted into 14 tubes, and 5 mL of sodium chloride solutions at a concentration ranging from 1% to 0.10% was added to each tube. The resulting suspensions were incubated for 30 min at room temperature and then centrifuged for 5 min at 1200× *g*. The intensity of haemolysis was evaluated in the supernatant using absorption spectrometry with a wavelength of 540 nm in each of the 14 test tubes. The highest concentration of saline at which haemolysis had just started was considered the minimum osmotic fragility, whereas the test tube with the lowest saline concentration at which haemolysis had finished was considered the maximum osmotic fragility. The osmotic fragility coefficient was calculated by comparing these two values.

Statistical analysis was performed using the IBM SPSS 23.0 (IBM SPSS Statistics, version 23.0, IBM Corp., Armonk, NY, USA) and R 3.4.3 (R Foundation for Statistical Computing, Vienna, Austria) software. The descriptive statistics for categorical and ordinal variables were frequency and percentage (%) and median and quartiles for quantitative variables. In all cases, two-way statistical criteria were used. The null hypothesis was rejected if *p* < 0.05.

The category frequencies of nominal variables were compared using the χ^2^ test or Fisher’s exact test, whereas the values of scale variables were compared using Student’s *t* test, and a univariate analysis of variance with subsequent Bonferroni pairwise comparison was used when the number of categories was greater than 2.

The predictive value of the laboratory tests for CSVD development was evaluated using ROC analysis and binary logistic regression, which was used to create a model for calculating the probability of CSVD development. Using the ROC curves for each parameter and the created predictive model, we determined the optimal threshold values, as well as their sensitivity and specificity.

## Figures and Tables

**Table 1:** Main demographic parameters and risk factors in patients with cerebral small vessel disease (CSVD) and in the control group.

Parameters	CSVD (*n* = 73)	Control (*n* = 19)	*p*
Age, years	60.1 ± 6.5	56.9 ± 6.4	0.061
Sex Women (*n*, %)	48 (65.8%)	14 (73.7%)	0.592
Arterial hypertension (AH) (*n*, %)	64 (87.7%)	9 (47.4%)	<0.001
Stage of AH (*n*, %)			
1	15 (20.5%)	5 (26.3%)	
2	13 (17.9%)	3 (15.8%)	
3	36 (49.3%)	1 (5.3%)	
Diabetes mellitus (DM) type 2 (*n*, %)	15 (20.5%)	0 (0.0%)	0.034
Hypercholesterolemia (total cholesterol >6.2 mmol/L or *statin use*) (*n*, %)	39 (53.4%)	9 (47.4%)	0.188
Smoking (*n*, %)	19 (26.0%)	8 (42.1%)	0.258
Obesity (body mass index >30 kg/m²) (*n*, %)	34 (46.6%)	5 (26.3%)	0.127

**Table 2:** Clinical symptoms and MRI signs of CSVD in patients with CSVD.

Parameters	CSVD (*n* = 73)
*Cognitive impairment* (*n*, %)*:*	73 (100%)
subjective	29 (39.7%)
mild	34 (46.6%)
dementia	10 (13.7%)
*Gait disturbances, unrelated to hemiparesis* (*n*, %)*:*	42 (57.5%)
mild	24 (32.8%)
moderate	7 (9.6%)
severe	11 (15.1%)
*Urinary disorders* (*n*, %)	27 (37.0%)
*History of stroke* (*n*, %)	15 (20.5%)
*WMH, Fazekas Scale* (*n*, %)	73 (100%)
grade 1	18 (24.7%)
grade 2	25 (34.2%)
grade 3	30 (41.1%)
*Lacunes* (*n*, %)	54 (73.9%)
*Microbleeds* (*n*, %)	45 (61.6%)
*Perivascular spaces* (*n*, %)	73 (100%)
>3 mm in the semiovale centres	4 (5.5%)
>3 mm in the basal ganglia region	22 (30.1%)

**Table 3:** Characteristics of the predictive model of CSVD development.

Predictors	B	*p*	OR	95% CI, Boundary	
				Lower	Upper
Salt sensitivity in 0.73% NaCl	−0.251	0.001	0.78	0.7	0.9
Minimum osmotic fragility	−9.833	0.001	0.02	0.005	0.5
Сonstant	6.306	0.001	547.7		

## Abbreviations

AH	Arterial hypertension
AUC	Area under the curve
BBB	Blood–brain barrier
CSVD	Cerebral small vessel disease
DM	Diabetes mellitus
MRI	Magnetic resonance imaging
ROC	Receiver operating characteristic
WMHs	White matter hyperintensities
